# Analgesic use among the Brazilian population: Results from the National Survey on Access, Use and Promotion of Rational Use of Medicines (PNAUM)

**DOI:** 10.1371/journal.pone.0214329

**Published:** 2019-03-21

**Authors:** Tatiane da Silva Dal Pizzol, Andréia Turmina Fontanella, Maria Beatriz Cardoso Ferreira, Andréa Dâmaso Bertoldi, Rogerio Boff Borges, Sotero Serrate Mengue

**Affiliations:** 1 Department of Production and Control of Medicines, School of Pharmacy, Federal University of Rio Grande do Sul (UFRGS), Porto Alegre, Rio Grande do Sul, Brazil; 2 Graduate Program in Epidemiology, School of Medicine, Federal University of Rio Grande do Sul (UFRGS), Porto Alegre, Rio Grande do Sul, Brazil; 3 Department of Pharmacology, Institute for Basic Health Sciences, Federal University of Rio Grande do Sul (UFRGS), Porto Alegre, Rio Grande do Sul, Brazil; 4 Graduate Program in Epidemiology, Federal University of Pelotas (UFPEL), Pelotas, Rio Grande do Sul, Brazil; Cleveland Clinic, UNITED STATES

## Abstract

**Purpose:**

To estimate the prevalence of use of analgesics in Brazil; and to characterize this use, according to sociodemographic and health-related characteristics.

**Methods:**

A cross-sectional population-based study (National Survey on Access, Use and Promotion of Rational Use of Medicines, PNAUM) was conducted between September 2013 and February 2014. A total of 41,433 people of all ages in Brazilian urban households were interviewed. Occasional use (within the last 15 days) and continuous use of non-opioid analgesics, opioid analgesics and non-steroidal anti-inflammatory drugs were investigated, regardless of whether this use occurred through prescription or self-medication. The main outcome was the use of at least one analgesic.

**Results:**

The majority of the individuals were female (52.8%), aged between 20 and 59 years (57.2%), with 1 to 8 years of schooling (45.6%). The overall prevalence of analgesic use was 22.8% [95% CI: 21.4–24.2]. The use of analgesics was significantly higher among women, adults and elderly (20 years or more), highly educated individuals and respondents who referred: diagnosis of one or more chronic diseases, using three or more medications, possession of health insurance and with one or more emergency care admittances or hospitalizations within the last year. Non-opioid analgesics were the agents most used (18.5% of the sample), followed by non-steroidal anti-inflammatory drugs (6.9%) and opioid analgesics (0.5%). The most commonly used drugs were metamizole (37.8% of all analgesics), paracetamol (25.3%) and diclofenac (10.7%). These drugs were used mainly to manage occasional health conditions, particularly pain.

**Conclusion:**

One in five Brazilians used some analgesic, especially non-opioid analgesics, to manage acute health problems such as painful conditions.

## Introduction

Pain is the most common complaint that leads individuals to seek healthcare services. Agents with analgesic action (including non-opioids, opioids and non-steroidal anti-inflammatory drugs [NSAIDs]) are among the most commonly used drugs for self-medication among the Brazilian population [1]. In Brazil and internationally, non-opioid analgesics and some non-steroidal anti-inflammatories are easy to acquire because of the affordable price and because they are sold without prescription.

Another factor contributing towards consumption of analgesics is that some analgesics also have therapeutic indications other than treatment of acute and chronic pain, such as management of fever (for which non-opioid analgesics and NSAIDs are used) and treatment of inflammatory conditions, such as temporomandibular joint arthritis (also using NSAIDs).

North American data have shown that pain medications stand out in both sales volume and volume of prescriptions. In 2014 and 2015, they were the third best-selling category in the USA, after cancer and antidiabetic agents [2,3]. In terms of number of prescriptions, they were the second most prescribed therapeutic category in 2016, only behind treatments for systemic arterial hypertension [4]. It is important to emphasize that studies have shown that the prevalence of non-prescription use is higher than use under prescription [5–7].

Population-based studies, with national samples estimating prevalence and characterizing users, the drugs most commonly used and the ways in which these drugs are used, are essential for assessing to what extent such use may represent a public health problem.

Studies of this nature have point out that analgesic and NSAID use occurs frequently, with higher prevalence of over-the-counter (OTC) drugs than of prescription medications [5–7]. However, no population-based studies published in Medline or EMBASE have been conducted in countries where the use of analgesics and NSAIDs that are subject to prescription (Rx) is facilitated by the practice of selling them in pharmacies without requiring presentation of the prescription. In this situation, the potential risks to which this population is subject may be even greater than those already pointed out in other scenarios.

Because of therapeutic importance of analgesics and their high consumption, the aim of the present study was to describe their use among the Brazilian population, with evaluation of: (a) the prevalence of general use and use in specific pharmacological groups; (b) the users’ profile, according to their sociodemographic and health-related characteristics; and (c) the frequency of use of specific drugs.

## Methods

### Sample

The present study was based on data collected through the National Survey on Access, Use and Promotion of Rational Use of Medicines (Pesquisa Nacional sobre Acesso, Utilização e Promoção do Uso Racional de Medicamentos no Brasil, PNAUM), which was a population-based cross-sectional study conducted in the urban areas of the five Brazilian regions (North, Northeast, Midwest, Southeast and South) between September 2013 and February 2014.

The study population comprised people of all ages residing in permanent private households, chosen in a complex survey with a probabilistic sample in three stages, in which the primary sampling unit corresponds to the municipalities, the second stage to the census sectors, and the third to domiciles. As the use of medicines varies according to age and gender, before starting the interview, these information of all household residents were recorded in order to identify who to be interviewed. The sample included eight demographic domains: (1) ages 0–4, both genders; (2) ages 5–19, both genders; (3) ages 20–39, female; (4) ages 20–39, male; (5) ages 40–59, female; (6) ages 40–59, male; (7) ages 60 or over, female; (8) ages 60 or over, male. That were replicated for each of the five Brazilian geographical regions.

Sample size was defined based on estimates of access and use of medicines obtained from previous surveys. By the end, PNAUM interviewed 41,433 people who, following adjustments by region, gender and age, represent approximately 171 million Brazilians living in urban areas of the country. More details on the PNAUM methods can be found elsewhere [8].

The survey was performed face-to-face. The research instruments were developed by researchers from seven universities in Brazil, having been standardized and tested previous to their implementation. The questionnaires included questions regarding the current use of medicines for chronic diseases and the use 15 days prior to the research to investigate signs, symptoms and acute conditions treated with medicines. The questions were adapted to be answered by the person responsible for the care of children (persons below 15 years of age) and those unable to communicate or self-report information due to physical or mental illness, speech impediment or lack of discernment to answer questions. The complete questionnaires can be seen on the PNAUM survey website (http://www.ufrgs.br/pnaum). The data were recorded on tablets, using software that had been developed specifically for the study.

The project was approved by the National Commission for Ethics in Research (*Comissão Nacional de Ética em Pesquisa*). All the participants signed two copies of the consent form before giving responses in the interview. The main person responsible for the children or incapable person, present in the face-to-face interview, gave informed consent and completed the interview.

### Data on participants and use of analgesics

We investigated the use of drugs to treat chronic diseases, based on information about previous diagnoses and medical indications for pharmacological therapy, along with the use of drugs to treat acute diseases or events, within the 15 days prior to the interview. The investigation on chronic diseases contained specific questions about high blood pressure, diabetes, heart diseases, hypercholesterolemia, stroke, lung disease, arthritis, arthrosis or rheumatism, depression and other chronic diseases lasting six months or longer. The investigation on acute diseases or events treated with medicines contained questions about the use of medications for the following conditions: infection, sleeping or anxiety problems, stomach or bowel problems, fever, pain, flu, cold or allergic rhinitis; along with use of vitamins, mineral supplements, appetite stimulants or tonics.

In the present study, we included occasional or continuous use of analgesics, irrespective of origin (through prescription or self-medication). The main outcome was the use of at least one analgesic (yes or no). The outcome variable considered the self-reported use of analgesics either for the treatment of chronic conditions or in the previous 15 days for the treatment of acute events or diseases.

Whenever possible, the drug names were copied from the packaging or prescription, to avoid misclassification. When no packaging or prescription was available, the names declared by the interviewees were recorded. The drugs were identified in the lists of the Brazilian Health Regulatory Agency (ANVISA) and were classified in accordance with the Anatomical Therapeutic Chemical (ATC) system [9].

The analysis was focused on the following ATC groups: M01A (non-steroidal anti-inflammatory), N02A (opioid analgesics) and N02B (other analgesics or antipyretics) [9]. In the present study, this last group was referred to as non-opioid analgesics, encompassing the following groups proposed in the ATC system: salicylic acid and derivatives, pyrazolones, anilides and other analgesics and antipyretics.

Combination drugs (products containing more than one analgesic ingredient) were categorized as a separate drug.

The most commonly reported drugs and their patterns of use were identified according to their use in treating acute or chronic conditions and the reasons for their use.

Sociodemographic and health-related variables were evaluated. The sociodemographic factors included the following: gender (female or male); age group (0 to 9; 10 to 19; 20 to 59; or ≥ 60 years); education (years completed) (never studied; 1 to 8; or > 8 years); and economic class (A/B; C; or D/E), in accordance with the Brazilian Economic Classification Criterion (*Critério de Classificação Econômica Brazil*, *CCEB*) of the Brazilian Association of Survey Companies (*Associação Brasileira das Empresas de Pesquisa*, *ABEP*) [10]. The economic classification took into account the conditions of the household, the number of goods acquired and the schooling of the person in charge of the household. The classes A1, A2, B1 and B2 represents high income, C1 and C2 represents medium income; and D and E represents low income. The health-related variables included the number of chronic diseases reported (none; 1; 2; or 3 or more); number of drugs in use (excluding analgesics) (none; 1; 2; 3 to 4; or 5 or more); health insurance coverage (yes or no); emergency visits within the previous 12 months (yes or no); and hospitalizations within the previous 12 months (yes or no).

Specifically among users of analgesics, use of multiple analgesics (i.e. concurrent use of more than one analgesic) was evaluated. For this, concomitant use of the following was assessed: (a) analgesics belonging to different groups; and (b) analgesics belonging to the same group. Additionally, the distribution of use of analgesics from the groups NO2B, NO2A and M01A, according to age group, was evaluated.

### Statistical analysis

Descriptive analyses were performed using SPSS version 18.0 for Windows (IBM SPSS Statistics, New York, USA).

An exploratory descriptive analysis on the variables was performed, with estimation of the prevalence of analgesic use (with 95% confidence interval), stratified according to each level of the sociodemographic and health-related variables. The chi-square test for independence was performed to evaluate possible associations. The significance level was set at 5% for all analyses.

## Results

The sociodemographic and health-related characteristics of the sample are presented in Table 1. The majority of the individuals were female (52.8%), aged between 20 and 59 years (57.2%), with 1 to 8 years of schooling (45.6%) and belonging to class C (55.3%). Approximately 69% did not have a chronic disease, 58.6% did not use drugs (excluding analgesics), 22% had health insurance coverage, 14.8% had received emergency care within the last 12 months and 5.9% had had one or more hospitalizations within the same period.

**Table 1:** Prevalence of analgesic use according to the sociodemographic and health-related characteristics of the sample. PNAUM, 2014 (n = 41,433).

		Sample	Prevalence of analgesic use		
		%	%^a^	95% CI	*P*^b^
Total		-	22.8	21.4–24.2	-
Gender	Male	47.2	17.9	16.5–19.3	<0.001
	Female	52.8	27.2	25.6–28.9	
Age (years)	0 to 9	13.7	13.9	12.4–15.6	<0.001
	10 to 19	16.0	15.7	13.6–17.9	
	20 to 59	57.2	25.1	23.5–26.8	
	≥ 60	13.2	30.0	28.8–32.4	
Education (years completed)	never studied	21.1	20.9	19.2–22.8	<0.001
	1 to 8	45.6	21.0	19.5–22.7	
	> 8	33.3	26.5	24.5–28.5	
Economic class^c^	A/B	22.3	23.0	20.8–25.3	0.413
	C	55.3	23.2	21.6–24.8	
	D/E	22.3	21.8	20.0–23.7	
Chronic diseases	None	69.1	17.1	15.9–18.4	<0.001
	1	17.0	28.5	26.3–30.8	
	2	7.2	36.8	34.1–39.7	
	≥ 3	6.6	52.6	49.8–55.4	
Number of drugs in use (excluding analgesics)	None	58.6	16.0	14.7–17.4	<0.001
	1	14.8	26.6	24.6–28.7	
	2	13.0	27.5	25.2–29.8	
	3 to 4	9.2	39.2	36.5–42.1	
	≥ 5	4.3	52.8	49.3–56.3	
Health insurance coverage	Yes	22.0	26.1	23.9–28.3	<0.001
	No	78.0	21.9	20.4–23.4	
Emergency visits^d^	Yes	14.8	42.4	40.2–44.7	<0.001
	No	85.2	19.4	18.1–20.8	
Hospitalizations ^d^	Yes	5.9	39.9	37.1–42.8	<0.001
	No	94.1	21.8	20.4–23.2	

^a^ Percentages adjusted according to sample weights and according to post-stratification (according to age and gender).

^b^ Chi-square test for independence.

^c^ According to the Brazilian Economic Classification Criteria 2013 (CCEB 2013) of the Brazilian Association of Research Companies (ABEP). Available at: http://www.abep.org

^d^ within the previous 12 months

CI: confidence interval

The overall prevalence of analgesic use was 22.8% (95% CI 21.4–24.2). Among adults (18 years or older), it was 25.9% (95% CI 24.4–27.5). The prevalence of use of non-opioid analgesics was 18.5% (95% CI 17.3–19.7), NSAIDs 6.9% (95% CI 6.4–7.5) and opioids 0.5% (95% CI 0.4–0.6). The prevalence of use was higher among women, individuals aged 60 years and over more and those with higher levels of schooling.

The prevalence was higher in the following situations: presence of chronic disease (irrespective of how many of them: from one to three or more); use of five or more drugs (other than analgesics), possession of health insurance coverage; use of emergency services within the last year; or occurrence of hospitalizations within the last year (Table 1).

**Fig 1:**
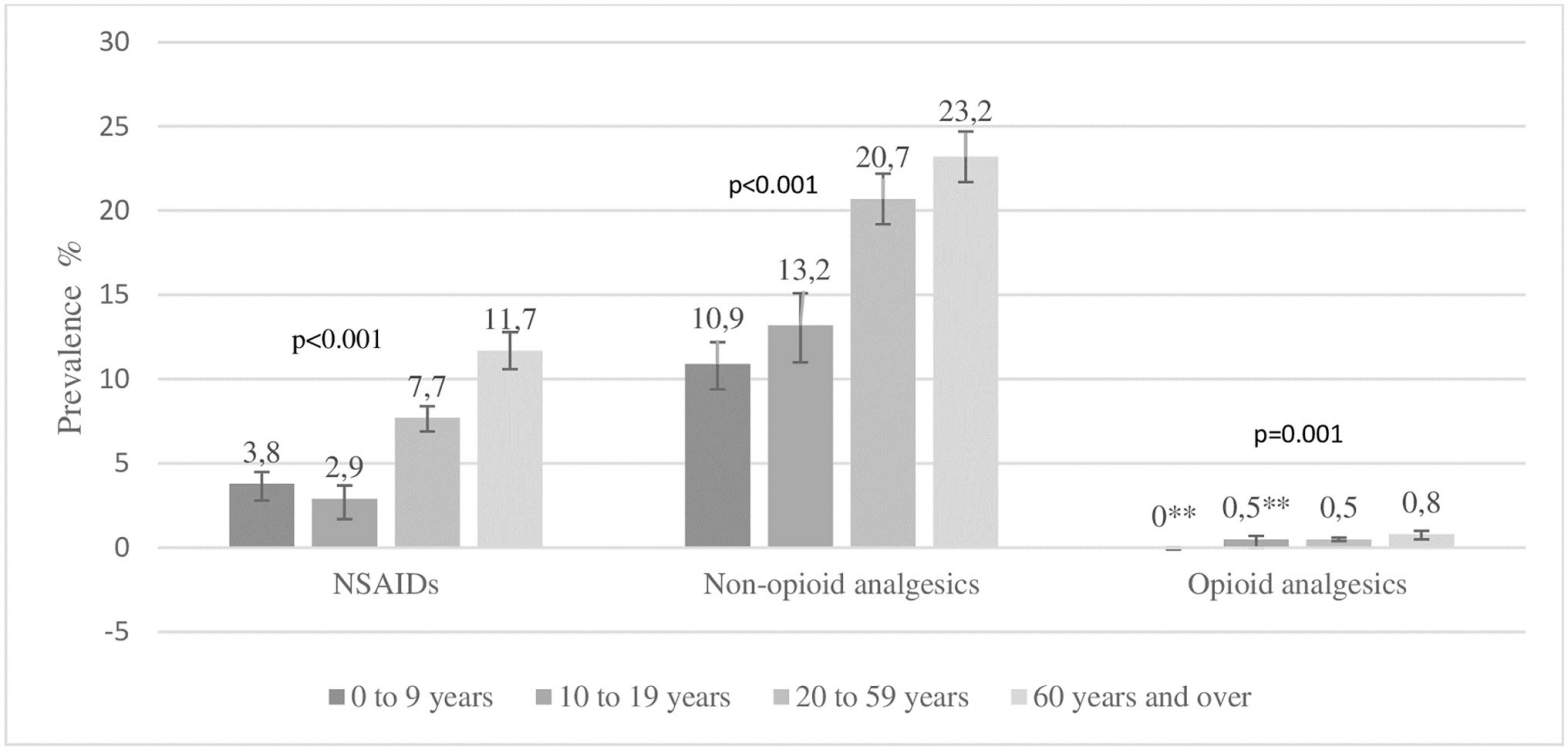
Prevalence of use of different groups of analgesics, categorized according to age group. PNAUM, 2014 (n = 41,433)*. * Percentages adjusted according to sample weights and according to post-stratification (according to age and gender). ** Coefficient of variation > 0.3. Caution is suggested in the interpretation.

The prevalence of use of the three groups of analgesics, categorized according to age group, is shown in Fig 1. In all of the three groups of analgesics, the prevalence of use was higher among individuals aged 60 years and over.

In a specific analysis that only included users of analgesics, most of these individuals reported consumption of only one analgesic (79.3%; 95% CI 77.8–80.7), while 16.8% (95% CI: 15.6–18.1) reported using two analgesics and 2.9% (95% CI 2.4–3.4) reported using three analgesics. 10.7% of these individuals used two or more drugs belonging to the same group (for example, two or more non-opioid analgesics).

**Fig 2:**
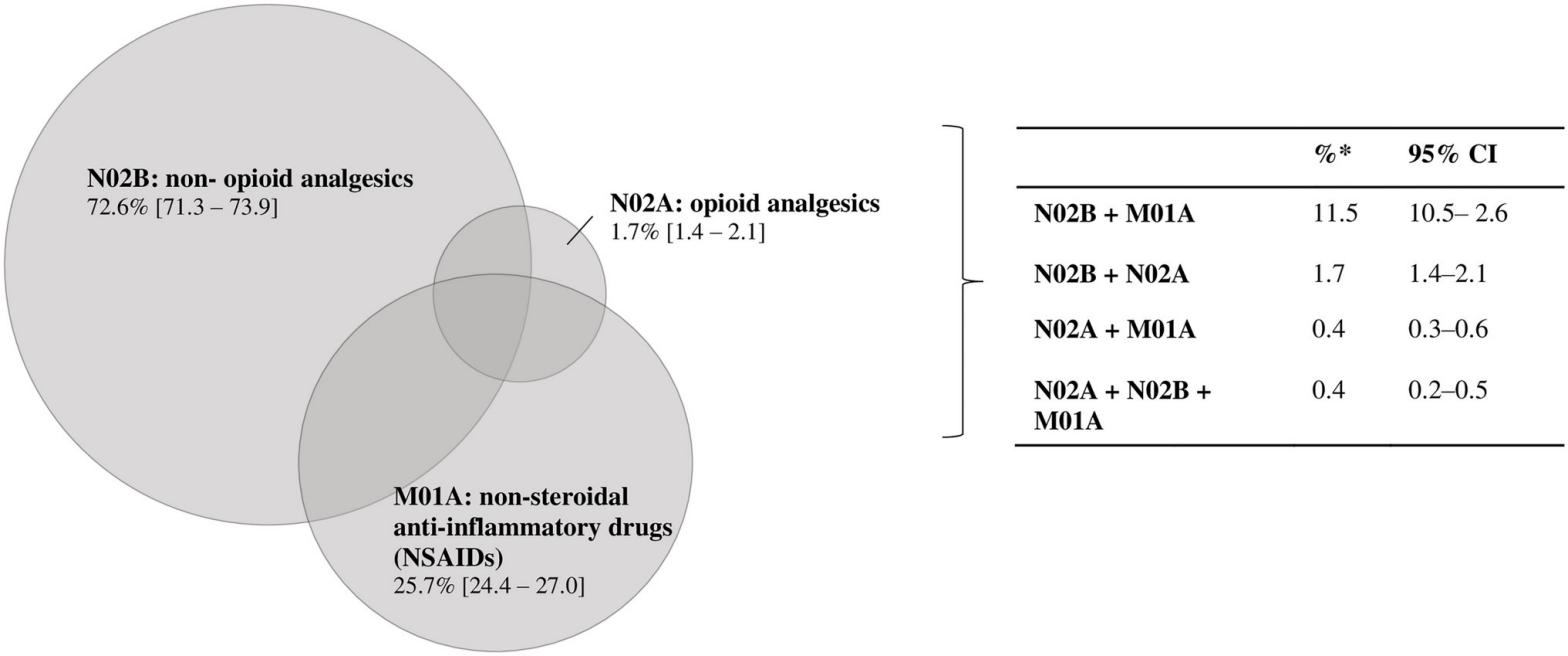
Frequency of analgesics used, according to the Anatomical Therapeutic Chemical (ATC) classification system, alone or in combination, by individuals who reported that they were using analgesics (n = 10,214). PNAUM, 2014*. * Percentages adjusted according to sample weights and according to post-stratification (according to age and gender).

The proportions of analgesics used alone or in combination by the individuals who cited the use of analgesics are shown in Fig 2.

Among all the analgesics used, the ones most commonly used were non-opioid agents (71.7%), followed by NSAIDs (26.6%) and opioid analgesics (1.7%); in each group, the drugs most consumed were, respectively, metamizole (52.8%), diclofenac (40.2%) and codeine (39.4%) (Table 2).

**Table 2:** Frequency of use of analgesics, according to the Anatomical Therapeutic Chemical (ATC) classification system, and the respective drugs most cited. PNAUM, 2014 (n = 13,054 analgesics).

Groups of analgesics/drugs	%	95% CI
**Non-opioid analgesics (N02B)**	**71.7** ^a^	**70.4–73.0**
metamizole	52.8	50.8–54.7
paracetamol	35.3	33.3–37.3
acetylsalicylic acid	11.9	10.9–13.1
**Non-steroidal anti-inflammatory (NSAIDs) (M01A)**	**26.6**	**25.3–27.9**
diclofenac	40.2	36.8–43.7
ibuprofen	24.6	22.1–27.2
nimesulide	13.6	11.8–15.6
**Opioid analgesics (N02A)**	**1.7**	**1.4–2.0**
codeine	39.4	31.1–48.4
papaverine	30.9	21.6–42.0
tramadol	25.7	18.4–34.6

^a^ The occurrence rate for propyphenazone (a non-opioid analgesic) was 0.04% and has not been represented in this table.

The ten most commonly used drugs are presented in Table 3. Metamizole, paracetamol and diclofenac accounted for 74% of all the analgesics used by the respondents.

**Table 3:** The ten most commonly used analgesics, regardless of Anatomical Therapeutic. Chemical (ATC) classification. PNAUM, 2014 (n = 13,054 analgesics).

Drugs	Frequency of use among the drugs mentioned	
	%	95% CI
metamizole	37.8	36.2–39.5
paracetamol	25.3	23.9–26.7
diclofenac	10.7	9.7–11.8
acetylsalicylic acid	8.5	7.8–9.4
ibuprofen	6.5	5.9–7.3
nimesulide	3.6	3.1–4.2
ketoprofen	1.0	0.7–1.3
meloxicam	0.9	0.7–1.2
codeine	0.7	0.5–0.9
piroxicam	0.6	0.4–0.9

Regarding health conditions, most of the interviewees used analgesics to treat acute diseases (90.2% [95% CI 89.2–91.2]), while 13.0% (95% CI 11.9–14.2) used them to treat chronic diseases. The pattern of analgesic use, in relation to chronic or occasional health conditions, motives and frequency of use, is presented in Table 4. Pain was the main reason cited for consumption, regardless of the group of analgesics.

**Table 4:** Pattern of use of analgesics, in relation to health condition and main reasons for use. PNAUM, 2014 (n = 13,054 analgesics).

	Non-opioid analgesics ^a^		Opioid analgesics ^b^		NSAIDs^c^	
		% (95% CI)		% (95% CI)		% (95% CI)
**Use for:**						
Chronic health conditions		10.4 (9.4–11.5)		29.1 (21.9–37.7)		21.3 (18.8–24.0)
Acute/occasional health conditions		89.6 (88.5–90.6)		70.9 (62.3–78.1)		78.7 (76.0–81.2)
**Main reasons for use**						
	Pain	63.1 (61.4–64.9)	Pain	62.1 (52.9–70.5)	Pain	56.4 (53.0–59.7)
	Other reasons	14.6 (13.4–15.9)	Other reasons	22.9 (16.2–31.2)	Other reasons	19.7 (17.3–22.4)
	Fever	13.8 (12.5–15.3)	Rheumatic disease	10.6 (7.0–15.8)	Infection	12.2 (10.4–14.3)
	Flu/cold	8.4 (7.5–9.5)	Infection	4.4 (2.0–9.5)*	Rheumatic disease	11.7 (9.9–13.7)

^a^ Acetylsalicylic acid, metamizole, paracetamol and propyphenazone (4 different drugs mentioned in this group)

^b^ Codeine, methadone, morphine, oxycodone, papaverine and tramadol (6 different drugs mentioned in this group)

^c^ Aceclofenac, mefenamic acid, benzydamine, celecoxib, ketoprofen, ketorolac, chondroitin, diacerein, diclofenac, etodolac, etoricoxib, phenylbutazone, flurbiprofen, glucosamine, ibuprofen, indomethacin, lornoxicam, meloxicam, naproxen, nimesulide, piroxicam and tenoxicam (22 different drugs mentioned in this group)

* Coefficient of variation > 0.3. Caution is suggested in the interpretation.

## Discussion

The prevalence of analgesic use in our study reveals that one in five Brazilians of all ages was using analgesics and that this use occurred mainly for treatment of occasional health conditions. The use was greater among women, adults and the elderly. Non-opioid analgesics were the most used, followed by NSAIDs. Opioids were infrequently used, compared with the other groups. Metamizole, paracetamol and diclofenac were among the most commonly used drugs, representing alone about three-quarters of all analgesics cited.

We did not find any previous studies with sufficient methodological similarities to make direct comparisons with estimates of overall prevalence of analgesic use. Among the differences between the studies, we can highlight the recall period for the evaluation of medicine use, the ages of the participants and the drugs analyzed. In our study, we evaluated current use or use within the 15 days prior to the interview, while other studies used periods of 7 days [6,11] or 30 days [5,7,12]. Previous studies found that different recall periods influenced the prevalence of occasional use of analgesics and NSAIDs, and the authors of those studies recommended the use of shorter reminder periods [13,14].

Women consumed more analgesics than men, and our finding was in line with the results from previous studies conducted in the USA [5] and in European countries [6,7,11,12,15]. The difference in use between men and women is due not only to biological differences (such as hormonal differences) and differences in the prevalence of certain pathological conditions (such as migraine, menstrual cramps and low back pain, among others) [16], but also to different behavior regarding health care [15,17].

The prevalence of use among adults and the elderly was higher, independently of the analgesic group. This finding was consistent with the epidemiological distribution of certain health conditions, such as muscle and back pain, headache and migraine, which occur more frequently in the economically active age range [18,19]. However, when the prevalence of use was stratified according to analgesic groups, the use of NSAIDs was found to be significantly higher among individuals aged 60 years and over, and the use of non-opioid analgesics was greater among those aged 20 years and over.

Use of analgesics was more frequent among people with higher schooling levels and among those with health insurance. This result may be related to greater access to healthcare services and medications.

Individuals with greater numbers of chronic diseases, those using polymedication and those who had been seen in emergency or hospitalized consultations within the last 12 months also presented higher prevalence of use. These results were consistent with the widespread use of analgesics to treat the painful and inflammatory conditions that are present in several chronic diseases; and with the presence of pain as part of the set of symptoms of various emergency or hospitalization situations.

Use of multiple analgesics (two or three) was observed among around 20% of the analgesic users. This percentage was higher than what was seen in a study in Scotland, in which the prevalence was found to be 4% [20]; but it was lower than what was found in an American study, in which the prevalence was close to 30% [5].

Higher consumption of non-opioid analgesics than of NSAIDs was also seen by Paulose-Ram et al in the USA [5], but not in a study conducted by Motola et al in Italy, in which the prevalence of NSAID use (M01) was twice the prevalence of non-opioid analgesic use [11], particularly regarding nimesulide.

The predominant use of non-opioid analgesics, particularly metamizole, paracetamol and acetylsalicylic acid, is consistent with their indication for very common acute and chronic pains, their widespread availability as over-the-counter medications and the fact that they are freely available in the public healthcare system. In addition, they are cheaper, have large use in the management of fever and are safer than other analgesics.

The consumption of metamizole by approximately half of the users of non-opioid analgesics is noteworthy. The adequacy of this drug remains a topic of discussion in the literature. Metamizole has been banned in many countries due to uncertainties regarding its safety, while in other countries it is still widely used, as is the case of Brazil [21,22].

NSAIDs, in turn, were consumed by 25.7% of the users of analgesics, and the drugs most commonly used were diclofenac, ibuprofen and nimesulide. We would suggest that the lower prevalence of NSAID use, compared with use of non-opioid analgesics, is appropriate, because although effective in managing various painful conditions, they present higher potential for adverse reactions. The NSAIDS that were most frequently used by respondents were those more popular, cheaper (particularly diclofenac) and available within the public healthcare system (especially ibuprofen). Diclofenac and ibuprofen are among the safest drugs in terms of frequency and severity of gastrointestinal adverse reactions, which are the most common adverse reactions of this therapeutic group [23,24].

Contrary to some estimates in the United States and other countries [25,26], the use of opioid analgesics in this Brazilian sample was low. Among the possible explanations for this difference, we can mention the low level of prescription of these drugs, which is more restricted to dental prescriptions of codeine (in association with paracetamol) and prescriptions issued by oncology services. This highlights the fact that in Brazil, services offering palliative care are still scarce. The low prevalence of use of opioid analgesics may also have been due to their lower acceptance among patients, given the stigma associated with their use in cancer and end-of-life situations, and also because of the higher cost of these drugs, compared with other analgesics. Lastly, unlike non-opioid analgesics and NSAIDs, dispensation of opioid drugs in Brazil is extensively controlled.

As shown in Fig 2, concomitant use of non-opioid analgesics and NSAIDs was prevalent, which may have been due to the various products containing these drugs that are available on the market and to use of combinations of two analgesics that happened to be available at home, as suggested by relatives, friends or neighbors, or through prescription. Overviews of Cochrane reviews have found that increased analgesic effectiveness is achieved through combination of paracetamol and ibuprofen, with Number Necessary to Treat (NNT) of less than two [27,28]. However, prior to using combinations of analgesics, care should be taken to ensure that each drug is safe and that the patient’s clinical condition allows such use. There is a need to assess the possible presence of risk factors and contraindications. In addition, the data from the Cochrane reviews cited above were obtained through administration of single doses, and use of higher doses may result in other outcomes. In addition, these reviews apply only to adult patients and to surgical procedures.

The present study has some limitations. The data relating to concomitant use of analgesics and use of two or more drugs containing the same analgesic ingredient should be interpreted with caution, since they refer to use of more than one analgesic within the period, but not necessarily at the same time (on the same day or at the same time of administration, for example). Therefore, it is not possible to conclude whether this concomitant use is inappropriate. Regarding acetylsalicylic acid, part of the use may have been due to its cardiovascular preventive effect and not necessarily to its analgesic effect. Lastly, PNAUM does not provide any data on pain recurrence and its management, since pain as a chronic health problem was not investigated through specific questions, unlike other chronic conditions addressed in the study (such as diabetes, hypertension and depression, among others).

Among the strengths of this study, it should be noted that, although self-reported data on drug use are susceptible to biases relating to social acceptability and respondents’ memory, population-based surveys tend to be more generalizable than are data originating from administrative prescribing or dispensing or from sales [29]. Thus, population-based data relate more closely to the actual consumption of medicines by the population and are not restricted to population groups served by a particular health insurance plan or specific healthcare programs. In addition, PNAUM was the first population-based survey conducted in Brazil that was specifically designed to evaluate Brazilians’ use of and access to drugs, based on a national sample that included individuals of all ages.

In conclusion, our study showed that analgesics, and especially non-opioid analgesics, are widely used by the Brazilian population to manage acute health problems such as painful conditions. The prevalence of use was higher among women, elderly people, individuals with higher schooling, those with greater numbers of chronic diseases, those using polymedication and those who, within the last 12 months, had been seen in emergency or hospitalized consultations. Future studies focusing on the use of NSAIDs among individuals at high risk of developing severe gastrointestinal, cardiovascular or renal adverse events are required, along with studies assessing differences in consumption between analgesic groups, according to location and form of acquisition (public or private).
