# Correction: Analgesic use among the Brazilian population: Results from the National Survey on Access, Use and Promotion of Rational Use of Medicines (PNAUM)

**DOI:** 10.1371/journal.pone.0216005

**Published:** 2019-04-18

**Authors:** Tatiane da Silva Dal Pizzol, Andréia Turmina Fontanella, Maria Beatriz Cardoso Ferreira, Andréa Dâmaso Bertoldi, Rogerio Boff Borges, Sotero Serrate Mengue

The following information is missing from the Funding statement: CAPES (Coordenação de Aperfeiçoamento de Pessoal de Nível Superior) assisted with the publication fee.
