# Correction: Analgesic use among the Brazilian population: Results from the National Survey on Access, Use and Promotion of Rational Use of Medicines (PNAUM)

**DOI:** 10.1371/journal.pone.0229039

**Published:** 2020-02-05

**Authors:** Tatiane da Silva Dal Pizzol, Andréia Turmina Fontanella, Maria Beatriz Cardoso Ferreira, Andréa Dâmaso Bertoldi, Rogerio Boff Borges, Sotero Serrate Mengue

The fourth author's initials appear incorrectly in the citation and are indexed incorrectly in PubMed. The correct initials are: Bertoldi AD. The correct citation is: da Silva Dal Pizzol T, Turmina Fontanella A, Cardoso Ferreira MB, Bertoldi AD, Boff Borges R, Serrate Mengue S (2019) Analgesic use among the Brazilian population: Results from the National Survey on Access, Use and Promotion of Rational Use of Medicines (PNAUM). PLoS ONE 14(3): e0214329. https://doi.org/10.1371/journal.pone.0214329
